# Identification of Mouse Mesenteric and Subcutaneous *in vitro* Adipogenic Cells

**DOI:** 10.1038/srep21041

**Published:** 2016-02-17

**Authors:** Yugo Miyata, Michio Otsuki, Shunbun Kita, Iichiro Shimomura

**Affiliations:** 1 Department of Metabolic Medicine, Graduate School of Medicine, Osaka University, Suita, Japan; 2 Department of Metabolism and Atherosclerosis, Graduate School of Medicine, Osaka University, Suita, Japan

## Abstract

Fat accumulation and the dysfunction of visceral white adipose tissue (WAT), but not subcutaneous WAT, cause abnormalities in whole body metabolic homeostasis. However, no current drugs specifically target visceral WAT. The primary reason for this is that a practical *in vitro* culture system for mesenteric adipocytes has not been established. To resolve this issue, we sought to identify *in vitro* adipogenic cells in mesenteric and subcutaneous WATs. First, we examined the expression pattern of surface antigens in stromal-vascular fraction (SVF) cells from mouse mesenteric and subcutaneous WATs, and found the expression of 30 stem cell-related surface antigens. Then, to evaluate the adipogenic ability of each fraction, we performed *in vitro* screening, and identified five candidate markers for mesenteric adipogenic cells and one candidate marker for subcutaneous adipogenic cells. To investigate whether *in vitro* adipogenic ability accurately reflects the conditions *in vivo*, we performed transplantation experiments, and identified CD9^−^ CD201^+^ Sca-1^−^ cells and CD90^+^ cells as mesenteric and subcutaneous *in vitro* adipogenic cells, respectively. Furthermore, mature adipocytes derived from mesenteric and subcutaneous adipogenic cells maintained each characteristic phenotype *in vitro*. Thus, our study should contribute to the development of a useful culture system for visceral adipocytes.

White adipose tissue (WAT) is anatomically classified into intra-abdominal visceral WAT and subcutaneous WAT^1^. Each form of WAT displays metabolically and biologically distinct features^2^,^3^ and has developmentally distinct precursor cells^4^. Visceral, but not subcutaneous, WAT fat accumulation and dysfunction cause abnormalities in whole body metabolic homeostasis resulting in life-threatening disorders^1^. Thus, targeted therapy against visceral WAT represents a potent rationale for ameliorating metabolic abnormalities. However, no drugs target molecules that are predominantly expressed, activated, or inactivated in visceral WAT. The primary reason for the lack of the drugs is that a practical and useful *in vitro* culture system for mesenteric adipocytes has not been established, causing difficulty in identifying novel drug targets using high-throughput screening^5^.

The strict definition of “visceral WAT” is the fat depot draining into the hepatic portal vein^1^. In human obesity, increased lipolysis in accumulated visceral WAT results in a greater release of free fatty acids into the portal vein, and exposes the liver to high concentrations of free fatty acids, causing metabolic abnormalities^1^,^6^. Although epididymal WAT has been frequently used as an alternative to visceral WAT in rodent models, epididymal WAT does not drain into the portal vein and are not anatomically comparable to visceral WAT in humans. Considering that previous studies have shown characteristic differences between epididymal and mesenteric WATs^7^,^8^,^9^, a more detailed analysis of mesenteric WAT should be required^10^.

There are cell culture models for the molecular analysis of adipocytes, including 3T3-L1, 3T3-F442, C3H-10T1/2, and Ob17^11^,^12^. These cell lines are derived from mouse embryos or epididymal WAT, which means they cannot be used to examine the function of distinct fat depots, such as visceral or subcutaneous WATs. Primary culture cells are another model type. Stromal-vascular fraction (SVF) cells in WAT include the cells that can differentiate into adipocytes in a culture dish (*in vitro* adipogenic cells), and these cells have been utilized in many studies^11^,^12^. However, the proportion of *in vitro* adipogenic cells in SVF varies by depots. SVF cells from visceral WAT have fewer *in vitro* adipogenic cells than those from subcutaneous WAT^13^,^14^. Due to the study limitations of mesenteric WAT, the molecular level biological differences between the two types of WAT have not yet been elucidated.

High-throughput screening in *in vitro* disease models is one of useful methods for discovering drug target genes or potential therapeutic compounds^5^,^15^. In adipocytes, anti-obesity drugs and genes related to metabolic disease were found through high-throughput screening using adipocyte cell lines^16^,^17^. However, adipocyte cell lines have different characters from WATs and primary adipocytes^11^,^12^,^18^,^19^. Therefore, an *in vitro* model of mesenteric adipocytes is necessary to identify novel type of drugs that target mesenteric adipocyte-specific molecules.

Here, we identified *in vitro* adipogenic cells in mesenteric and subcutaneous WATs. Our *in vitro* experiments and a subsequent *in vivo* study demonstrate that the surface antigens CD9^−^, CD201^+^, and Sca-1^−^ represent specific markers of *in vitro* adipogenic cells in mesenteric WATs, whereas CD90^+^ specifically marks *in vitro* adipogenic cells in subcutaneous WATs. Furthermore, mature adipocytes derived from mesenteric and subcutaneous adipogenic cells maintained each characteristic phenotype *in vitro*, as reported for each WAT in *ex vivo* and *in vivo* experiments^8^,^20^,^21^.

## Results

### *In vitro* screening for adipogenic cells identifies candidate markers

**Figure 1:**
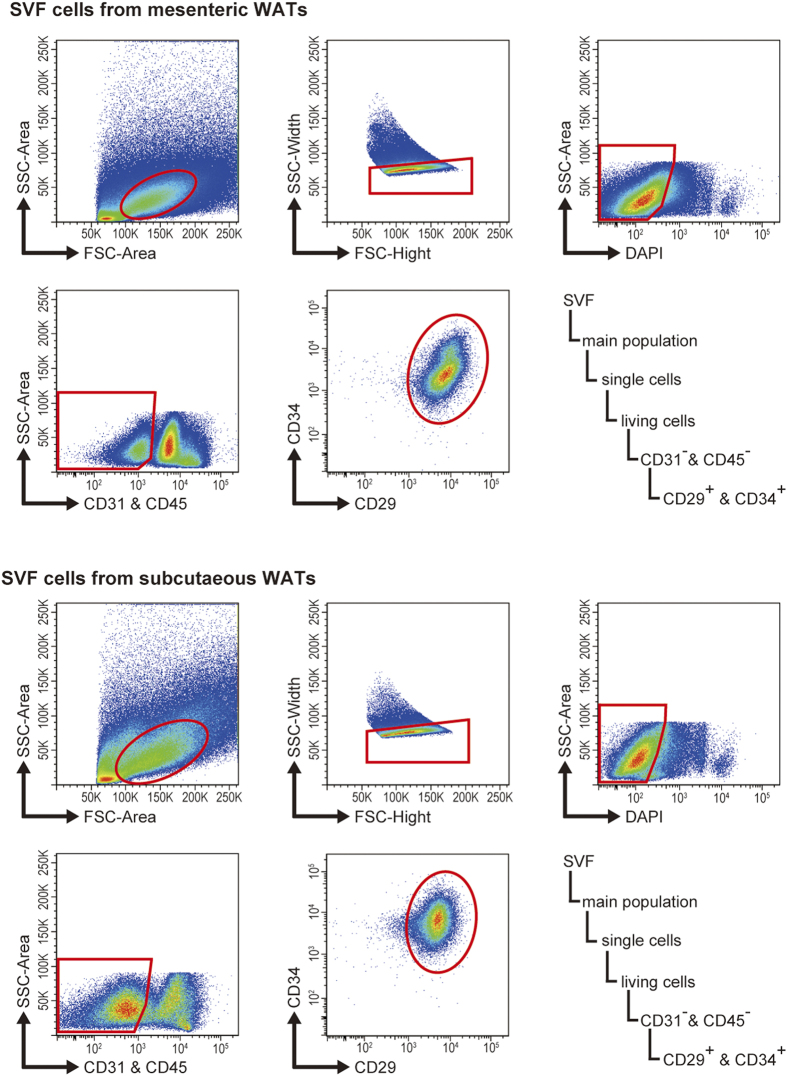
Gating hierarchies and dot plot images of SVF cells from mesenteric and subcutaneous WATs. The fraction of Lin^−^ CD29^+^ CD34^+^ cells was used in the current study.

To identify *in vitro* adipogenic cell markers in mesenteric and subcutaneous WATs, we initially attempted to clarify the expression pattern of surface antigens in freshly isolated SVF cells derived from each WAT. To ensure the inclusion of surface markers of various stem/progenitor cells such as embryonic stem cells, hematopoietic stem cells, and mesenchymal stem cells, we selected 103 molecules that were categorised as stem cell-related surface antigens in catalogues provided by the following companies: BD Biosciences, eBioscience, BioLegend, Abcam, and Beckman Coulter (Table 1 and Supplementary Dataset S1). Freshly isolated SVF cells from mesenteric and subcutaneous WATs were gated into Lin^−^ CD29^+^ CD34^+^ fibroblasts according to a previous report^22^, and antigen expression was tested in this fraction (Fig. 1). We then selected antigens that were expressed in >5% of Lin^−^ CD29^+^ CD34^+^ fibroblasts (Table 1, the antigens in bold italic style, and Supplementary Fig. S1). Nearly all (>95%) of the Lin^−^ CD29^+^ CD34^+^ cells expressed CD44, CD49e, CD51, and CD140α (PDGFRα). Therefore, we excluded these antigens from subsequent experiments (Supplementary Fig. S1). As SSEA-3 was not expressed in Lin^−^ CD29^+^ CD34^+^ fibroblasts from mesenteric WAT, this antigen was assessed only in cells derived from subcutaneous WAT (Supplementary Fig. S1-2).

To determine whether the selected antigens represented surface markers for *in vitro* adipogenic cells, we sorted Lin^−^ CD29^+^ CD34^+^ cells into antigen-positive and antigen-negative fractions, cultured the cells, and compared their differentiation abilities *in vitro*. The following two criteria were used to determine whether a molecule was an *in vitro* adipogenic cell marker: i.) a 1.5-fold increase in triglyceride accumulation between the antigen-positive and antigen-negative fractions, as quantified by the measurement of Oil Red O dye, and ii.) a 10-fold increase in the adiponectin concentrations in the media between the antigen-negative and antigen-positive fractions. Adiponectin is exclusively expressed in and secreted from mature adipocytes^1^; therefore it is used as a specific mature adipocyte marker^23^,^24^,^25^. We considered surface antigens that fulfilled both criteria as candidate adipogenic cell markers. As shown in Supplementary Fig. S2, CD9^−^, CD38^+^, CD201^+^, BP-1^+^, and Sca-1^−^ fractions from mesenteric WAT showed more than a 1.5-fold higher value of Oil Red O staining and more than a 10-fold higher adiponectin concentration, whereas the same fractions from subcutaneous WAT did not fulfill the aforementioned criteria (Supplementary Fig. S2). Only CD90^+^ cells in subcutaneous WAT met the criteria, whereas CD90^+^ cells in mesenteric WAT did not meet the criteria (Supplementary Fig. S2-2). The remaining antigens were excluded from the pool of candidates by these criteria (Supplementary Fig. S2).

### CD9, CD38, CD201, BP-1, and Sca-1 in mesenteric WAT, and CD90 in subcutaneous WAT, are candidate markers for *in vitro* adipogenic cells

**Figure 2:**
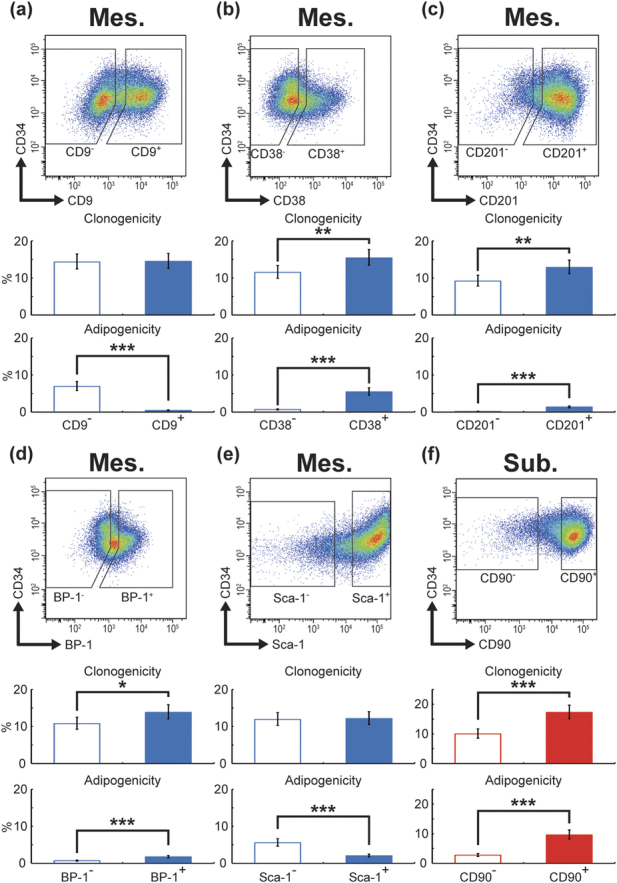
Clonogenicity and adipogenicity of the candidate surface markers of mesenteric and subcutaneous *in vitro* adipogenic cells. SVF cells from mesenteric WAT (**a–e**) or subcutaneous WAT (**f**) were sorted, seeded on 96 well plates at the desired concentration, cultured. After reaching 100% confluency, the cells were treated with adipogenic differentiation medium. The medium was replaced with DMEM containing 10% FBS 2 days after commencement of differentiation; after 48 h, Oil Red O staining was performed and colony- and adipocyte-positive wells were counted. The fraction of Lin^−^ CD29^+^ CD34^+^ cells is shown in the dot plot pictures. The gating hierarchy of Lin^−^ CD29^+^ CD34^+^ cells is shown in Fig. 1. The raw data are shown in Supplementary Dataset S2. The values are expressed as the means ± SD. **p < 0.01; ***p < 0.001. n = 3 in each group.

To confirm the adipogenic ability of the selected fractions (Supplementary Fig. S2), the clonogenicity and adipogenicity of each fraction were measured. As described in Fig. 2a,e (raw data in Supplementary Dataset S2), CD9^−^ and Sca-1^−^ cells from mesenteric WAT exhibited significantly higher adipogenicity as compared with CD9^+^ and Sca-1^+^ cells, respectively, whereas no differences in clonogenicity were found. CD38^+^, CD201^+^, and BP-1^+^ cells from mesenteric WAT exhibited 1.3-, 1.4-, and 1.3-hold higher clonogenicity than CD38^−^, CD201^−^, and BP-1^−^ cells, respectively. Those positive fractions also exhibited 7.9-, 13.0-, and 2.6-fold higher adipogenicity than their counter fractions (Fig. 2b–d; raw data in Supplementary Dataset S2). Similarly, the clonogenicity and adipogenicity of CD90^+^ cells from subcutaneous WAT were 1.7- and 3.4-hold higher than CD90^−^ cells, respectively (Fig. 2f; raw data in Supplementary Dataset S2).

**Figure 3:**
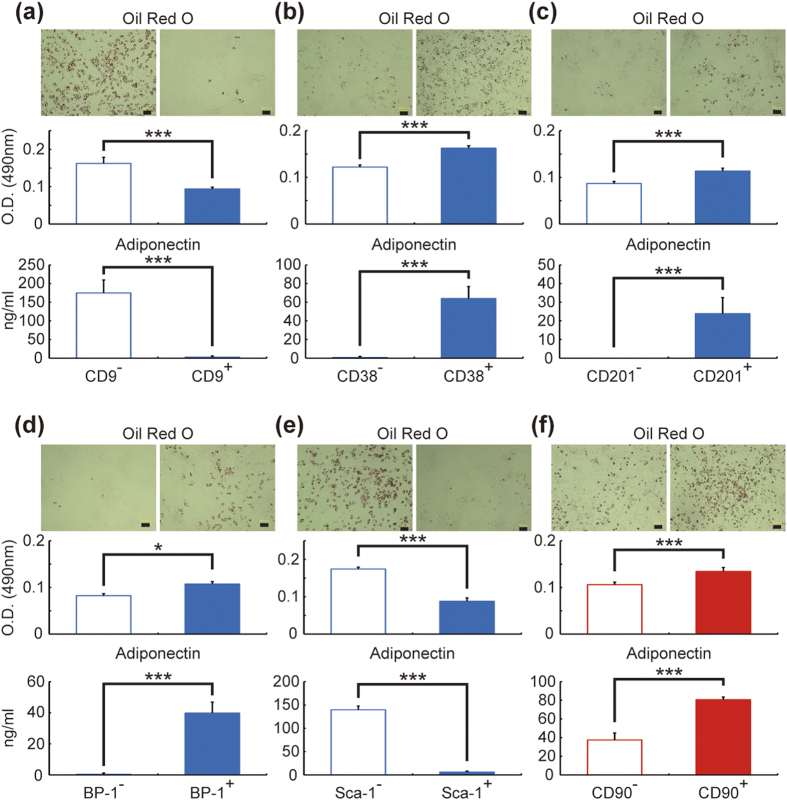
CFU-F-matched experiment for validating the candidate surface markers of mesenteric and subcutaneous *in vitro* adipogenic cells. SVF cells from mesenteric WAT (**a–e**) or subcutaneous WAT (**f**) were sorted, seeded at the same CFU-Fs, cultured and, after reaching 100% confluency, treated with adipogenic differentiation medium. The medium was replaced with DMEM containing 10% FBS 2 days after commencement of differentiation; after 48 h, the medium was collected and Oil Red O staining was performed. The adiponectin concentrations in the medium were assessed. Scale bars, 100 μm. The values are expressed as the means ± SD. *p < 0.05; ***p < 0.001. n = 4–5 in each group.

Differences in clonogenicity were found in some fractions, which raises the possibility that the high adipogenic ability shown in *in vitro* screening depends on differences in clonogenicity (Supplementary Fig. S2). Thus, we next performed colony forming unit-fibroblast (CFU-F)-matched experiments (Fig. 3). Significantly higher values of Oil Red O staining and adiponectin concentrations were found in CD9^−^, CD38^+^, CD201^+^, BP-1^+^, and Sca-1^−^ cells from mesenteric WAT than in each counter fraction (Fig. 3a–e). Similarly, CD90^+^ cells from subcutaneous WAT showed a higher value of Oil Red O staining and adiponectin concentrations than CD90^−^ cells (Fig. 3f). Collectively, CD9, CD38, CD201, BP-1, and Sca-1 were found as candidate markers for *in vitro* adipogenic cells in mesenteric WAT, and CD90 was found as a candidate marker for *in vitro* adipogenic cells in subcutaneous WAT.

### CD9^**−**^ CD201^+^ Sca-1^**−**^ cells from mesenteric WAT and CD90^+^ cells from subcutaneous WAT have high adipogenic ability *in vivo*

**Figure 4:**
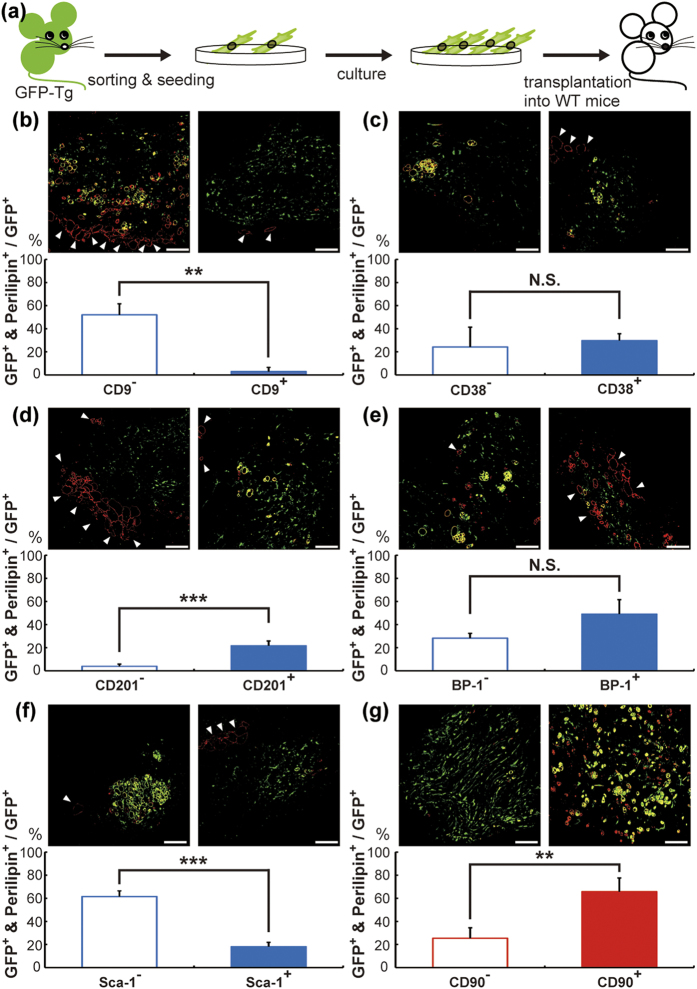
*In vivo* adipogenic ability of the candidate *in vitro* adipogenic cells. The scheme of the transplantation experiment is shown in (**a**). SVF cells from GFP-Tg mice were sorted and cultured. The cells were then detached, suspended in Matrigel and transplanted into WT mice. The cells from mesenteric WAT were injected into mesenteric WAT (**b–f**), and those from subcutaneous WAT were injected subcutaneously (**g**). Injected Matrigel was harvested one week after transplantation and subjected to the immunoflorescence assay. Perilipin protein immunostaining was performed to detect adipocytes. GFP and perilipin are shown in green and red, respectively. White arrow heads point to endogenous adipocytes. DAPI staining of each image was shown in Supplementary Fig. S3. Scale bars, 100 μm. The values are expressed as the means ± SD. **p < 0.01; ***p < 0.001; N.S., not significant. n = 3-4 in each group.

To investigate whether *in vitro* adipogenic cells certainly have adipogenic ability *in vivo*, a transplantation experiment was performed. Lin^-^ CD29^+^ CD34^+^ cells from GFP-Tg mice were sorted and cultured at up to 60%−80% confluence (typically this took for 3−5 days). Subsequently, the cells were detached, suspended in Matrigel, and transplanted into wild type mice. Cells from mesenteric WAT were injected into mesenteric WAT, and those from subcutaneous WAT were injected subcutaneously (Fig. 4a). One week later, the Matrigel was harvested and subjected to immunohistochemical analysis, where we assessed the rate of GFP^+^ adipocytes for total exogenous cells. The adipocyte number was counted by evaluating expression of perilipin, a known lipid droplet-associated protein and an adipocyte marker^26^. As shown in Fig. 4b, 52.1% of CD9^−^ cells from mesenteric WAT differentiated into adipocytes, whereas CD9^+^ cells seldom differentiated (2.9%, Fig. 4b). Similarly, CD201^+^ and Sca-1^−^ cells showed higher differentiation potential *in vivo* compared with CD201^−^ and Sca-1^+^ cells, respectively (CD201, 3.8% vs. 21.6%; Sca-1, 61.5% vs. 18.0%; Fig. 4d,f). However, no significant differences were observed in CD38 and BP-1 cells (Fig. 4c,e). CD90^+^ cells from subcutaneous WAT differentiated into adipocytes more frequently than CD90^−^ cells (Fig. 4g). These data show that mesenteric CD9^−^, CD201^+^, and Sca-1^−^ cells and subcutaneous CD90^+^ cells, having high adipogenicity *in vitro*, also have ability to differentiate into mature adipocytes *in vivo*.

**Figure 5:**
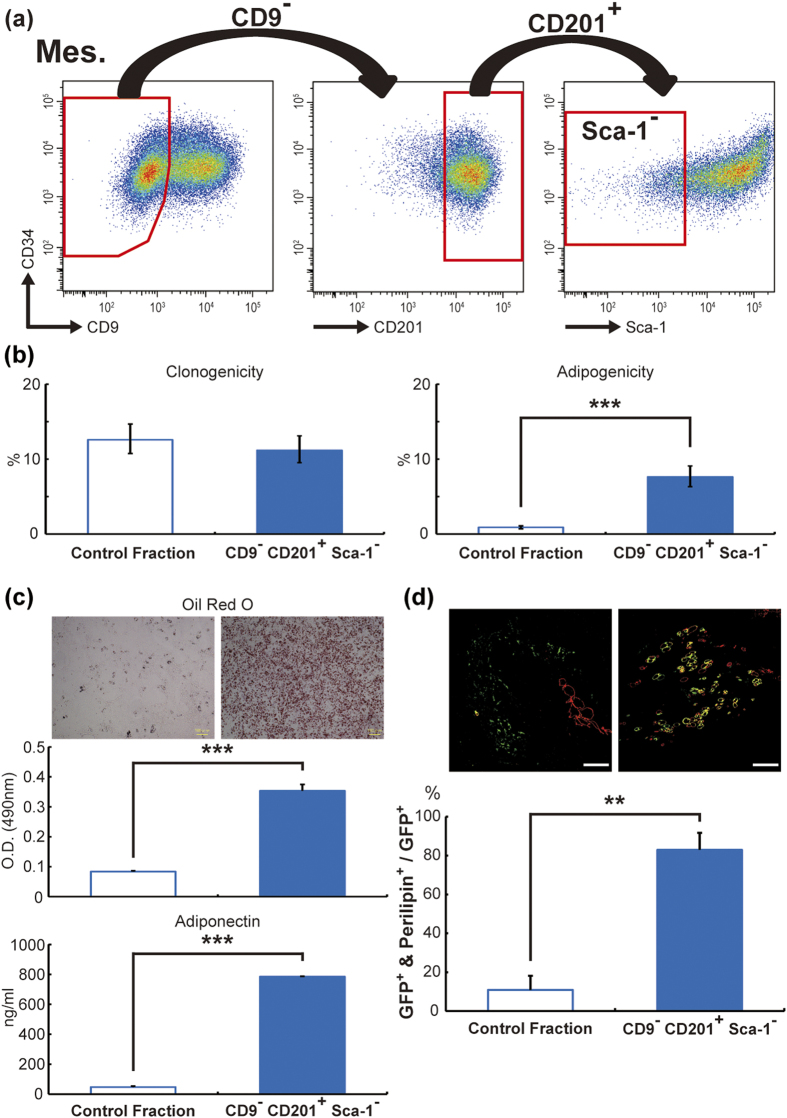
Identification of specific surface markers of mesenteric *in vitro* adipogenic cells. Dot plot images of CD9^−^ CD201^+^ Sca-1^−^ cells are shown in (**a**). CD9^−^ CD201^+^ Sca-1^−^ cells from SVF cells of mesenteric WAT were subjected to *in vitro* assays ((**b)** clonogenicity and adipogenicity assays, see Fig. 2 and raw data in Supplementary Dataset S2; (**c**) Oil Red O staining and adiponectin secretion under CFU-F-matched conditions, see Fig. 3) and *in vivo* assay (**d**, transplantation experiments, see Fig. 4; DAPI staining was shown in Supplementary Fig. S3). The term “Control Fraction” indicates CD29^+^ CD34^+^ cells, excluding CD9^−^ CD201^+^ Sca-1^−^ cells. Scale bars, 100 μm. The values are expressed as the means ± SD. **p < 0.01, ***p < 0.001, n = 3 (**b,c**), n = 5 and 3 (**d**) in each group.

To confirm the adipogenic ability of CD9^−^ CD201^+^ Sca-1^−^ cells from mesenteric WAT, we first compared the clonogenicity and adipogenicity of these cells with those of control fraction cells (CD29^+^ CD34^+^ cells excluding CD9^−^ CD201^+^ Sca-1^−^ cells) (Fig. 5a,b). CD9^−^ CD201^+^ Sca-1^−^ cells and the control fraction cells showed no significant differences in terms of clonogenicity, but the former cells had higher adipogenicity than the latter ones (Fig. 5b; raw data in Supplementary Dataset S2). Furthermore, CD9^−^ CD201^+^ Sca-1^−^ cells showed significantly higher Oil Red O staining and adiponectin concentrations in the media in CFU-F-matched *in vitro* assay (Fig. 5c). CD9^−^ CD201^+^ Sca-1^−^ cells also showed a 7.6-fold increase in *in vivo* adipogenic ability compared with control cells (Fig. 5d).

**Figure 6:**
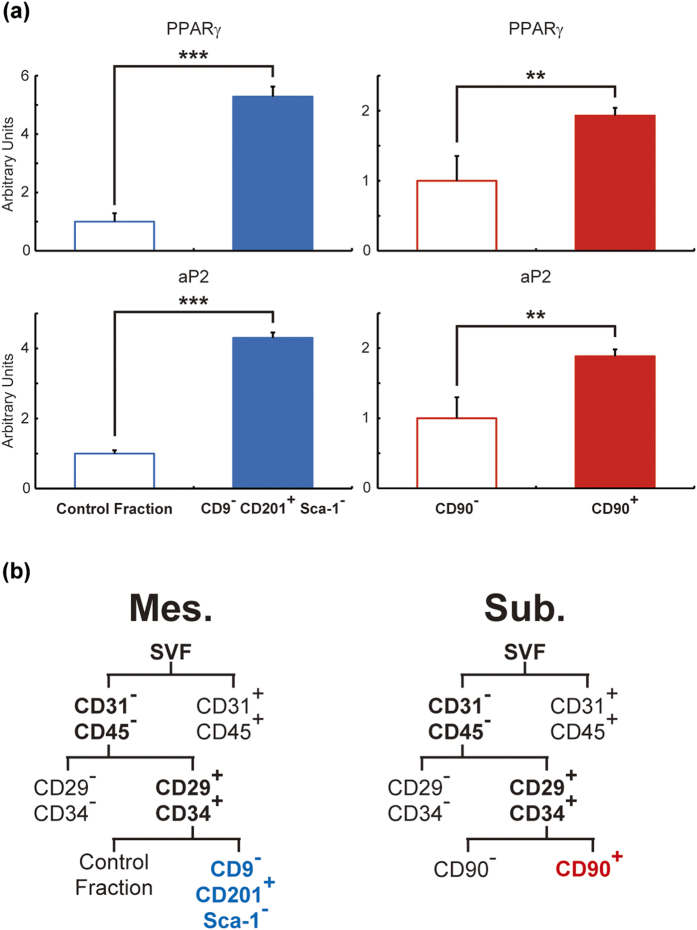
Gene expression levels of adipocyte markers in differentiated adipocytes derived from mesenteric and subcutaneous *in vitro* adipogenic cells. Gene expression levels of PPARγ and aP2 were measured in mature adipocytes derived from mesenteric and subcutaneous adipogenic cells *in vitro* (**a**). Gating hierarchies of mesenteric and subcutaneous *in vitro* adipogenic cells are shown in (**b**). The values are expressed as the means ± SD. **p < 0.01, ***p < 0.001, n = 3–5 in each group.

In addition to adiponectin secretion and Oil Red O staining, mRNA expressions of adipocyte marker genes were measured in differentiated CD9^−^ CD201^+^ Sca-1^−^ cells and CD90^+^ cells (Fig. 6a). Mesenteric CD9^−^ CD201^+^ Sca-1^−^ cells and subcutaneous CD90^+^ cells showed higher PPARγ and aP2 mRNA expressions than each counter fraction. Collectively, CD9^−^, CD201^+^, and Sca-1^−^ were identified as specific markers for mesenteric *in vitro* adipogenic cells which can also differentiate *in vivo*. Similarly, CD90^+^ was identified as a specific marker of subcutaneous *in vitro* adipogenic cells which can also differentiate *in vivo* (Fig. 6b).

### Differentiated adipocytes from mesenteric and subcutaneous *in vitro* adipogenic cells have different features

**Figure 7:**
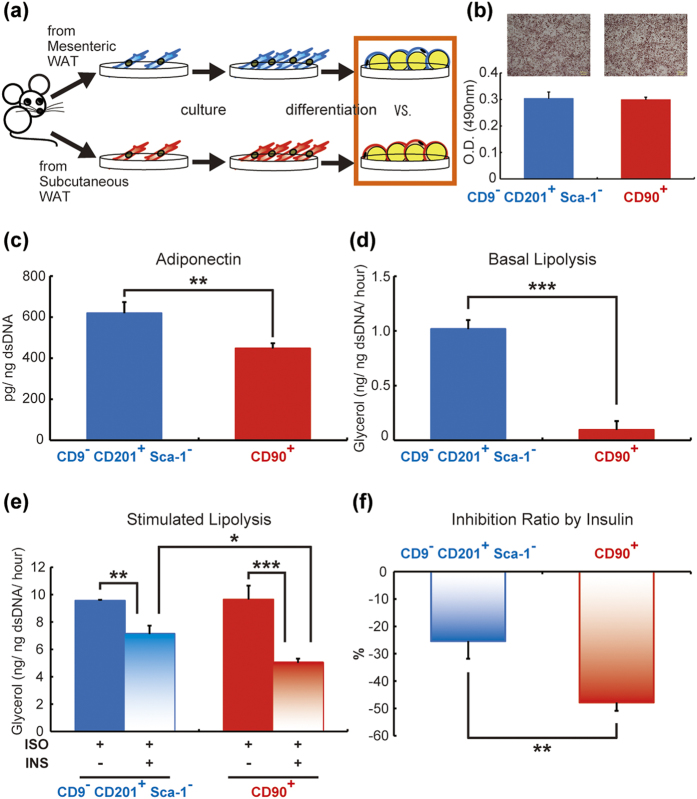
Distinct features of cultured mature adipocytes derived from mesenteric and subcutaneous *in vitro* adipogenic cells. The experimental scheme is shown in (**a**). Oil Red O staining was performed in differentiated *in vitro* adipogenic cells (**b**). Under the condition of the same differentiation efficiency in the two *in vitro* adipogenic cells, adiponectin secretion (**c**), basal lipolytic activity (**d**), stimulated lipolytic activity (**e**), and the inhibition ratio of lipolytic activity by insulin (**f**) were evaluated in mature adipocytes derived from mesenteric and subcutaneous adipogenic cells *in vitro*. ISO, isoproterenol; INS, insulin. The values are expressed as the means ± SD. *p < 0.05, **p < 0.01, ***p < 0.001, n = 3 in each group.

According to previous observations of different features between mesenteric and subcutaneous WATs^27^,^28^, we attempted to validate whether differentiated adipocytes derived from mesenteric and subcutaneous adipogenic cells maintained each characteristic phenotype *in vitro* (Fig. 7a). We first confirmed differentiation efficiency in mesenteric and subcutaneous *in vitro* adipogenic cells. As shown in Fig. 7b, no significant difference was observed in Oil Red O staining between the two differentiated cells (Fig. 7b). Under the condition of the same differentiation efficiency in the two *in vitro* adipogenic cells, higher adiponectin production was observed in mesenteric adipocytes compared with subcutaneous adipocytes *in vitro* (Fig. 7c); similar results were previously observed in our *ex vivo* experiments^20^ and in another group’s *in vivo* experiments^21^. We then compared lipolytic activities between two distinct adipocytes according to the previous *ex vivo* study^8^. The basal lipolytic activity was higher in mesenteric adipocytes than in subcutaneous adipocytes (Fig. 7d). Under isoproterenol-stimulated conditions, the lipolytic activity of subcutaneous adipocytes was more inhibited by insulin than that of mesenteric adipocytes (Fig. 7e). The inhibition rates of the lipolytic activity in mesenteric and subcutaneous adipocytes were 25.4% and 47.9%, respectively (Fig. 7f). Taken together, we identified mesenteric and subcutaneous *in vitro* adipogenic cells in mesenteric and subcutaneous WATs, respectively. We also confirmed that adipocytes from each *in vitro* adipogenic cell displayed different physiological features.

## Discussion

Although many have reported different features between visceral and subcutaneous WATs, and they also differ in their association with metabolic abnormalities and diseases^1^,^2^,^3^, the mechanisms underlying these differences remain unclear. The present study identified surface markers for *in vitro* adipogenic cells in mesenteric and subcutaneous WATs, and reproduced two types of functionally unique adipocytes using a common culture method. Thus, our data should contribute to the development of an *in vitro* mesenteric adipocyte model, which may facilitate the production of new types of drug targeting visceral WAT dysfunction through a gain- or loss-of-function approach and *in vitro* high-throughput screening^5^.

Small number of mesenteric *in vitro* adipogenic cells in mesenteric WAT have prevented studies on detailed mechanisms of the functional difference between mesenteric and subcutaneous adipocytes. In our study, a new method for sorting the *in vitro* adipogenic cells was found, and most of the cells were differentiated into adipocytes (Figs 5c and 7b). To our knowledge, no studies have reported such high differentiation efficiency in mesenteric adipocytes. Although there are several critical tips to yield maximum differentiation efficiency (see Methods) and several technical problems to construct a more useful culture system (see below), our findings should be the first step toward molecular biological analysis in mesenteric adipocytes.

To identify *in vitro* adipogenic cells, we used a conventional culture method for pre-adipocyte cell lines, which had two stages; a proliferative step, where cells were cultured with growth medium up to 100% confluency, and an induction step, where cells were treated with differentiation medium. In our experience, the highest adipogenic efficiency was obtained when cells were induced within four days after sorting and seeding. However, the longer the sorted cells were cultured as the growth step, the lower their differentiation efficiency, as described previously^29^. Indeed, in the adipogenicity assay, sorted cells were grown for two weeks prior to differentiation, and adipogenicity was less than 10% (Figs 2 and 5b, Supplementary Dataset S2). To establish a useful culture system which allows cell proliferation without loss of adipogenic ability, refining the conventional culture system is required from the perspective of soluble factors^30^ and culture substratum^31^.

A major study limitation in our experiment is the lack of the characterization of mature adipocytes differentiated from transplanted cells (Figs 4 and 5d). We used immunohistochemical method to characterize only transplanted cells (GFP^+^ cells), because many host cells (GFP^−^ cells) migrated into injected Matrigel, and a whole Matrigel analysis does not represent transplanted donor cells (GFP^+^ cells) (Supplementary Fig. S3). At first, according to the previous findings of depot-specific genes in epididymal and subcutaneous WATs^32^, we tested whether these gene expressions could also be drastically different between mesenteric and subcutaneous WATs. Among these genes, Tbx15, Shox2, and En1 showed striking difference in their gene expression between mesenteric and subcutaneous WATs (Supplementary Fig. S4). Further, antibodies against mouse Tbx15 (Abcam, #ab55740) or mouse Shox2 (Novus, #NB100-92302), which could be used in our experiment system, were commercially available, and then we tried to detect the proteins in the transplanted cells. However, we failed to detect Tbx15 and Shox2 expressions in our samples (data not shown). Further study should be required, and this process should be helpful to construct the culture system reflecting *in vivo* environment.

Although we showed that mesenteric CD9^−^ CD201^+^ Sca-1^−^ cells and subcutaneous CD90^+^ cells had the ability to differentiate into mature adipocytes both *in vitro* and *in vivo*, recent study suggested that various types of adipocyte precursor cells existed in a single WAT depot *in vivo*^33^. To delineate the contribution of the identified fractions in adipocyte precursor cells *in vivo*, we performed a direct transplantation experiment, which omits the culturing step of sorted cells before transplantation, and was thought to represent actual contributions of isolated fractions in adipocyte differentiation *in vivo*. Because most Lin^−^ cells in SVF cells are positive for CD29 and CD34 (>95%, Fig. 1), the fraction should include most *in vivo* adipocyte precursor cells. Therefore, we first examined the rate of *in vivo* adipocyte precursor cells in CD29^+^ CD34^+^ cells (the parental fraction of *in vitro* adipogenic cells, Fig. 6a). Freshly isolated CD29^+^ CD34^+^ cells were transplanted *in vivo* without culture, and their differentiation rate was evaluated. We found that approximately 90% of CD29^+^ CD34^+^ cells differentiated into adipocytes *in vivo* in both mesenteric and subcutaneous WATs (data not shown). Considering that 8% and 65% of *in vitro* adipogenic cells were contained in CD29^+^ CD34^+^ cells in mesenteric and subcutaneous WATs, respectively (Figs 5a and 2f), other cell fractions should also have the potency to differentiate into adipocytes *in vivo*. Thus, mesenteric CD9^−^ CD201^+^ Sca-1^−^ cells and subcutaneous CD90^+^ cells should be some but not all of *in vivo* adipocyte precursor cells in each WAT depot, although only these fractions have high adipogenic ability in a conventional culturing and differentiation method used in this study.

In our experiments, neither CD24 nor PDGFRβ (CD140β) was shown to be a specific marker for subcutaneous *in vitro* adipogenic cells; these data differ from previous reports^22^,^26^,^34^ (Supplementary Figs S2-1 and S2-3). We speculate that the difference can be attributed to the age of the mice used in each study. To identify adult adipogenic cells, 10- to 12-week-old mice were used in the current study, whereas 30-day-old and 42-day-old mice were used in studies by Tang and Berry^26^,^34^, respectively. Because i.) fat mass and body fat content in mice increase during the first 10 weeks of life and are maintained or slightly increased thereafter^29^,^35^, ii.) adipocyte progenitor cells in 4- to 6-week-old mice have a different role from those of 10- to 12-week-old mice^36^, and iii.) adipocyte turnover occurs throughout life in WAT^37^, different adipogenic cells may exist in young (<10-week-old) and fully mature adult (>10-week-old) stages^36^. In the current study, we used 10- to 12-week-old mice because we sought to specifically identify adult adipogenic cells.

Joe *et al.* also showed that Sca-1^+^ cells were adipocyte precursor cells^38^. In their study, clonogenicity and adipogenicity of Sca-1^+^ cells were higher than Sca-1^−^ cells in subcutaneous and epididymal or parametrial WATs. However, their data had very low numerical values (Sca-1^−^ vs Sca-1^+^; 0.4% vs 7% in clonogenicity and 0.06% vs 2.4% in adipogenicity in subcutaneous WAT, 0.25% vs 2% in clonogenicity and almost 0% vs 0.1% in epididymal or parametrial WATs). Our experiment showed >10% and 0.5% of clonogenicity and adipogenicity in most fractions, respectively (Fig. 2 and raw data in Supplementary Dataset S2). This discrepancy may be due to the protocols of the clonogenicity and adipogenicity assays. Joe et al. simultaneously sorted and seeded using the “clone sorting mode” of the FACSAria system, while we followed the typical protocol and its precautions (please see Nature Protocols^39^, and Methods). We sorted cells using the “4-way sorting mode” to avoid contamination and then seeded the sorted cells soon after counting live cells in order to seed an accurate number of cells^39^ (during cell count, we checked that the cells were dispersed in a single cell). Another reason we adopted the latter protocol is fatal cell damage during sorting. According to BD Biosciences customer service, turbulence flow is generated in a flow cell, which can result in severer damage to cells passing through the machine. In fact, we observed that the number of living cells was smaller than that of FACS-counting cells, and the collection rate of living cells was also lower in negative fractions of almost all surface markers than in the positive fractions. Therefore, in the previous report^38^, measurement of clonogenicity and adipogenicity based on FACS-counting cells might be underestimated, and a higher adipogenicity in Sca-1^+^ cells could be attributed to higher clonogenicity. In our study, to exclude the possibility that high clonogenicity represents high adipogenicity, we performed CFU-F-matched experiments (Figs 3 and 5c). This validation process clearly showed that the Sca-1^−^ fraction included more *in vitro* adipogenic cells than the Sca-1^+^ fraction.

Sca-1 is widely known as a specific marker of hematopoietic stem cells^40^ and bone marrow mesenchymal stem cells^41^, and has a function related to stemness in these cells^42^,^43^. Accordingly, the Sca-1^+^ fraction was predicted to contain more adipogenic cells and was used as a source of adipogenic cells from WATs^22^,^30^,^34^. However, no studies have yet directly compared adipogenic ability between Sca-1^−^ and Sca-1^+^ cells in SVF cells in WATs, excluding the aforementioned study by Joe^38^. Here we demonstrated that Sca-1^−^ cells in mesenteric WAT included more *in vitro* adipogenic cells than Sca-1^+^ cells (Figs 2e and 3e), and Sca-1^−^ and Sca-1^+^ cells in subcutaneous WAT did not show a marked difference in adipogenic ability (Supplementary Fig. S2-4). It is controversial whether all progenitor cells express Sca-1; for example, in the pancreas and testes, progenitor cells do not express Sca-1^44^,^45^. In this context, our findings may warn against usage of Sca-1 expression as an adipogenic cell marker. In addition, our data show that the Sca-1 protein does not have a functional role in adipogenesis.

## Methods

### Animals

Male FVB/NJcl mice were purchased from CLEA Japan (Tokyo, Japan). GFP-Tg mice were purchased from SLC (Tokyo, Japan) and were backcrossed with FVB/NJcl more than 7 times. Heterozygous GFP-Tg mice were used in the experiments because some of the homozygotes were small and short-lived, whereas the heterozygotes had similar body weights and lengths as the WT mice. The experimental protocols were approved by the Ethics Review Committee for Animal Experimentation of Osaka University, Graduate School of Medicine. All experiments on animals were carried out in accordance with the approved guidelines.

### Isolation of SVF cells

SVF cells of mesenteric and subcutaneous WATs from 10- to 12-week-old mice were isolated as described earlier^46^ with a slight modification. Collagenase type II (Sigma, St. Louis, MO) and DNase I (Roche, Indianapolis, IN) were used to final concentrations of 400 U/ml and 0.1 mg/ml, respectively.

### Flow cytometry and multi-colour sorting

Isolated SVF cells were incubated with TruStain fcX (BioLegend, San Diego, CA) or 2.4 G2 (TONBO Biosciences, San Diego, CA) for 7 min. The cells were then rinsed and resuspended in phenol red-free DMEM-10% FBS and stained with the primary antibodies (Supplementary Dataset S1). For cells from GFP-Tg mice, florescent streptavidin conjugates were used as secondary detection reagents (Supplementary Dataset S1). Following these reactions, the cells were rinsed 2 or 3 times and suspended in phenol red-free DMEM-10% FBS and analyzed or sorted using a FACSAria II cell sorter that is equipped with a 4-laser and 14-colour system (BD Biosciences, Franklin Lakes, NJ). To protect the cells from mechanical damage and death, sample agitation was set to 200 rpm or turned off.

### Cell culture and differentiation

Sorted cells were cultured on a collagen I-coated plate and proliferated in DMEM-10% FBS supplemented with 10 ng/ml murine basic-FGF (Peprotech, Rocky Hill, HJ)^22^. Twelve to 24 h after reaching 100% confluency, the cells from subcutaneous WAT were treated for 2 days with differentiation medium, 10% FBS-supplemented DMEM containing 1 μM of insulin (Nacalai Tesque, Kyoto, Japan), 0.5 mM 1-methyl-3-isobutyl-xanthine (Nacalai Tesque), and 1 μM dexamethasone (Nacalai Tesque). For the cells from mesenteric WAT, 10 μM pioglitazone, a PPARγ agonist, was added to the differentiation medium because the cells could not otherwise differentiate into adipocytes. To assess *in vitro* differentiation ability, an equal number of antigen-positive and antigen-negative cells or CFU-Fs was seeded, and the cells were cultured at up to 100% confluency and then treated with differentiation medium within 4 days after seeding. Differentiated cells were further maintained in DMEM-10% FBS. To accurately examine *in vitro* differentiation ability, the cells of a certain fraction and its counter fraction were cultured in the same way, and induced at the same time.

### Oil Red O staining and quantification

As described above, sorted cells were treated with differentiation medium. Two days later, the medium was replaced with DMEM-10% FBS. The cells were cultured for 2 more days before they were fixed with 10% formaldehyde (Nacalai Tesque) for 10 min. The fixed cells were washed twice with PBS and with 60% isopropanol for 1 min, and incubated in Oil Red O (Nacalai Tesque) solution in 60% isopropanol for 15 min. Following the removal of the Oil Red O solution, the stained cells were washed once with 60% isopropanol and twice with PBS. PBS was added to each well, and images of stained cells were acquired using a BioZero microscope BZ-9000 (Keyence, Osaka, Japan). To quantify the Oil Red O staining, the PBS was discarded, and the wells were completely dried and incubated with 100% isopropanol for 15 min with gentle agitation. The OD at 490 nm was measured.

### Measurement of adiponectin concentration

For *in vitro* screening (Supplementary Fig. S2) and CFU-F-matched experiments (Figs 3 and 5b), sorted cells were treated with differentiation medium for 2 days, and the medium was replaced with DMEM containing 10% FBS. After 48 h of incubation, the culture medium was collected, and the adiponectin concentrations were measured with an adiponectin ELISA kit (Otsuka, Tokyo, Japan). To compare the adiponectin secretion abilities of adipocytes derived from mesenteric CD9^−^ CD201^+^ Sca-1^−^ cells and subcutaneous CD90^+^ cells (Fig. 6c), the sorted cells were treated with differentiation medium containing 10 μM pioglitazone.

### Clonogenicity and adipogenicity

Clonogenicity and adipogenicity were measured using the limiting dilution method according to the typical protocol and its precautions about two critical steps; counting cells and diluting the cell suspension^39^. To avoid contamination, cells were sorted using “4-way sorting mode” in the FACSAria system. Although FACS cell sorters count the number of sorted cells, it is not always correct due to fetal cell damage during sorting, according to BD Biosciences customer service. Therefore, we used not “clone sorting mode” but “4-way sorting mode”. Living cells were counted and diluted into the desired seeding concentration soon before seeding on 96 well plates. During counting cells, we checked that the cells were dispersed in a single cell. Seeded cells were cultured in DMEM-10% FBS with murine basic-FGF for 2 weeks, and then treated with differentiation medium for 2 days. Differentiated cells were maintained with DMEM-10% for further 2 days. At day 4, cells were fixed and subjected to Oil Red O staining. At least 40 replicate wells were generated for each cell dose (raw data in Supplementary Dataset S2). Limiting dilution analysis calculations were based on the single hit Poisson model (see Statistics)^38^,^47^.

### Transplantation experiments

SVF cells from GFP-Tg mice were sorted and cultured. When the cells expanded at up to 60–80% confluency (typically this took 3–5 days) in DMEM-10% FBS with basic-FGF, they were detached with Accutase (Innovative Cell Technologies, San Diego, CA) and suspended in Matrigel (BD Biosciences) before implantation. The cells from subcutaneous and mesenteric WATs were injected subcutaneously and into mesenteric WATs of 14- to 20-week-old male wild-type mice, respectively, because mesenteric *in vitro* adipogenic cells were not differentiated when injected subcutaneously. For mesenteric injection, Matrigel was solidified by warming right after injection to prevent leakage. A section of mesenteric WAT adjacent to the mesenteric lymph node in 14- to 20-week-old mice has sufficient thickness for Matrigel injection. One week after transplantation, the injected Matrigel was harvested and fixed with 10% formaldehyde. A stereomicroscope was used when injected Matrigel was collected from mesenteric WAT, and the WAT surrounding Matrigel were trimmed away. The sample was then subjected to immunoflorescence staining.

### Immunoflorescence stainingh

Fixed Matrigel was immersed in 90% ethanol overnight before it was paraffin-embedded. Paraffin sections were de-waxed and rehydrated by serial immersion in xylene, ethanol, and water. Antigens were retrieved by boiling the slides in HistoVT ONE (Nacalai Tesque) for 20 min, followed by incubation at RT for 20 min. The slides were blocked with PBS-10% normal donkey serum (Jackson Immuno Research, West Grove, PA) for 60 min and then incubated overnight with goat anti-GFP polyclonal antibody (1:100; ab6673, Abcam, Cambridge, MA) and rabbit anti-perilipin monoclonal antibody (1:250; #9349, Cell Signaling Technology, Danvers, MA) in PBS-1% BSA. Chicken anti-goat IgG Alexa Fluor 647 (1:200; Invitrogen, Carlsbad, CA) and donkey anti-rabbit IgG Alexa Fluor 555 (1:200; Invitrogen) were used as secondary antibodies. Following the secondary antibody reactions, the sections were washed and incubated with goat anti-chicken IgG Alexa Fluor 647 (1:200; Invitrogen) for 60 min. The sections were then rinsed, counterstained with DAPI (Invitrogen), and mounted in CC Mount (Diagnostic BioSystems, Pleasanton, CA). Florescence was visualized using a FluoView confocal laser-scanning microscope (Olympus, Tokyo, Japan).

### RNA extraction and quantitative real-time PCR

Total RNA was extracted using RNeasy micro kit (Qiagen, Valencia, CA). First-strand cDNA was synthesized from total RNA using the Transcriptor First Strand cDNA Synthesis Kit (Roche, Indianapolis, IN). Real-time PCR was performed on LightCycler system using the FastStart DNA Master SYBR Green I (Roche), and values are normalized to the level of 36B4 mRNA. Sequences of primers used for real-time PCR were the following: PPARγ, 5′-ATC TTA ACT GCC GGA TCC ACA A-3′ and 5′-GCC CAA ACC TGA TGG CAT T-3′; aP2, 5′-CCG CAG ACG ACA GGA-3′ and 5′-CTC ATG CCC TTT CAT AAA CT-3′; 36B4, 5′-GCT CCA AGC AGA TGC AGC A-3′ and 5′-CCG GAT GTG AGG CAG CAG-3′.

### Lipolysis assay

Sorted cells were induced with differentiation medium containing 10 μM pioglitazone and maintained in DMEM-10% FBS containing 100 nM insulin (Nacalai Tesque) and 10 μM pioglitazone until day 4.5. Primary adipocytes, especially adipocytes from cells in mesenteric WAT, displayed high lipolytic activity even under basal conditions (Fig. 6d), and their lipid contents were reduced without insulin. To maximise the lipid content of the adipocytes, 10 μM pioglitazone was added to the maintenance medium. Before the lipolysis assays were conducted, the medium was washed out and replaced with DMEM-10% FBS without insulin and pioglitazone at day 4.5, and the cells were incubated for 12 h. To measure the basal lipolytic activity, the cells were washed and incubated with KRBH buffer −4% albumin. After 4 h of incubation, the medium was collected, and the glycerol concentrations were measured with an adipolysis assay kit (Cayman Chemical Company, Ann Arbor, MI). As an internal control, the double-stranded DNA content of the cells was quantified using a CellTox green assay kit (Promega, Fitchburg, WI). To measure the stimulated lipolytic activity and inhibitory effect of insulin on lipolysis, the cells were treated with 1 μM isoproterenol (Sigma-Aldrich, St. Louis, MO) in the absence or presence of 1 μM insulin for 2 h.

### Statistical Analysis

All data were expressed as mean ± SD. Differences between two groups were examined for statistical significance by the Student’s t-test in Figs 3, 4, 5c,d, 6a and 7b–d,f. Differences among four groups in Fig. 7e were examined by ANOVA followed by Tukey-Kramer test. A P value <0.05 denoted the presence of a statistically significant difference. The JMP Pro 11.0.0 software (SAS Institute. Inc., Cary, NC) was used for these analyses. In clonogenicity and adipogenicity assays (Figs 2 and 5b), the statistically-significant differences were examined by the Pearson’s *chi-square* test, using ELDA software of the Walter and Eliza Hall Institute of Medical Research, Melbourne, Australia (http://bioinf.wehi.edu.au/software/limdil/)^47^.

## Additional Information

**How to cite this article**: Miyata, Y. *et al.* Identification of Mouse Mesenteric and Subcutaneous *in vitro* Adipogenic Cells. *Sci. Rep.* **6**, 21041; doi: 10.1038/srep21041 (2016).

## Supplementary Material

Supplementary Information

Supplementary Dataset S1

Supplementary Dataset S2

## Figures and Tables

**Table 1:** Stem cell-related cell surface antigens.

CD2	CD36	***CD81***	CD133	***CD317***	***SSEA-3***
CD3	***CD38***	CD84	CD135	CD324	SSEA-4
CD4	CD40	CD86	***CD140a***	CD326	Tim-3
***CD9***	CD41	CD88	***CD140b***	CD339	
CD11a	CD43	***CD90***	CD144	***BP-1***	
CD11b	***CD44***	CD93	CD146	c-Met	
CD11c	CD47	CD101	CD150	Dlk	
***CD13***	CD48	***CD105***	***CD155***	DLL1	
CD14	CD49b	***CD106***	***CD166***	DLL4	
CD15	CD49d	***CD107a***	CD183	EGFR	
CD18	***CD49e***	***CD107b***	CD184	Endomucin	
CD19	***CD49f***	CD109	CD195	ESAM	
***CD20***	***CD51***	CD115	***CD201***	integrin beta 7	
CD21/CD35	***CD54***	CD117	***CD202b***	Jagged 2	
***CD24***	***CD55***	CD119	CD226	Ly-6G/Ly-6C	
CD25	CD56	CD120a	CD229	Notch-1	
***CD26***	CD59	CD120b	CD271	***Notch-2***	
CD27	CD71	CD121a	CD282	Notch-3	
CD28	***CD73***	CD123	CD284	Notch-4	
CD30	CD79a	CD127	CD309	***Sca-1***	

All the molecules were categorised as stem cell-related antigens in the catalogues provided by the following companies: BD Biosciences, eBiosciences, BioLegend, Abcam, and Beckman Coulter.

Surface antigens expressed in CD29^+^ CD34^+^ cells are shown in bold italic style.
