# Supplementary material for: Identification of Mouse Mesenteric and Subcutaneous in vitro Adipogenic Cells
Source: Sci Rep. 2016 Feb 17;6:21041. doi: 10.1038/srep21041 (PMC4756711; doi:10.1038/srep21041)
Supplement: Supplementary Information [file srep21041-s1.pdf]

Identification of Mouse Mesenteric and Subcutaneous *in vitro* Adipogenic Cells

Yugo Miyata[1,*], Michio Otsuki[1,*], Shunbun Kita[1,2], and Iichiro Shimomura[1]

[1]Department of Metabolic Medicine, Graduate School of Medicine, Osaka University, Suita, Japan

[2]Department of Metabolism and Atherosclerosis, Graduate School of Medicine, Osaka University, Suita, Japan

Corresponding authors:

Yugo Miyata, PhD

Department of Metabolic Medicine, Graduate School of Medicine, Osaka University

2-2 B5, Yamadaoka, Suita, Osaka 565-0871, JAPAN

Tel: +81-6-6879-3742, Fax: +81-6-6879-3739

E-mail: yugo-miyatavic@fbs.osaka-u.ac.jp

yugomiyata@gmail.com

Michio Otsuki, MD, PhD

Department of Metabolic Medicine, Graduate School of Medicine, Osaka University

2-2 B5, Yamadaoka, Suita, Osaka 565-0871, JAPAN

Tel: +81-6-6879-3732, Fax: +81-6-6879-3739

E-mail: otsuki@endmet.med.osaka-u.ac.jp

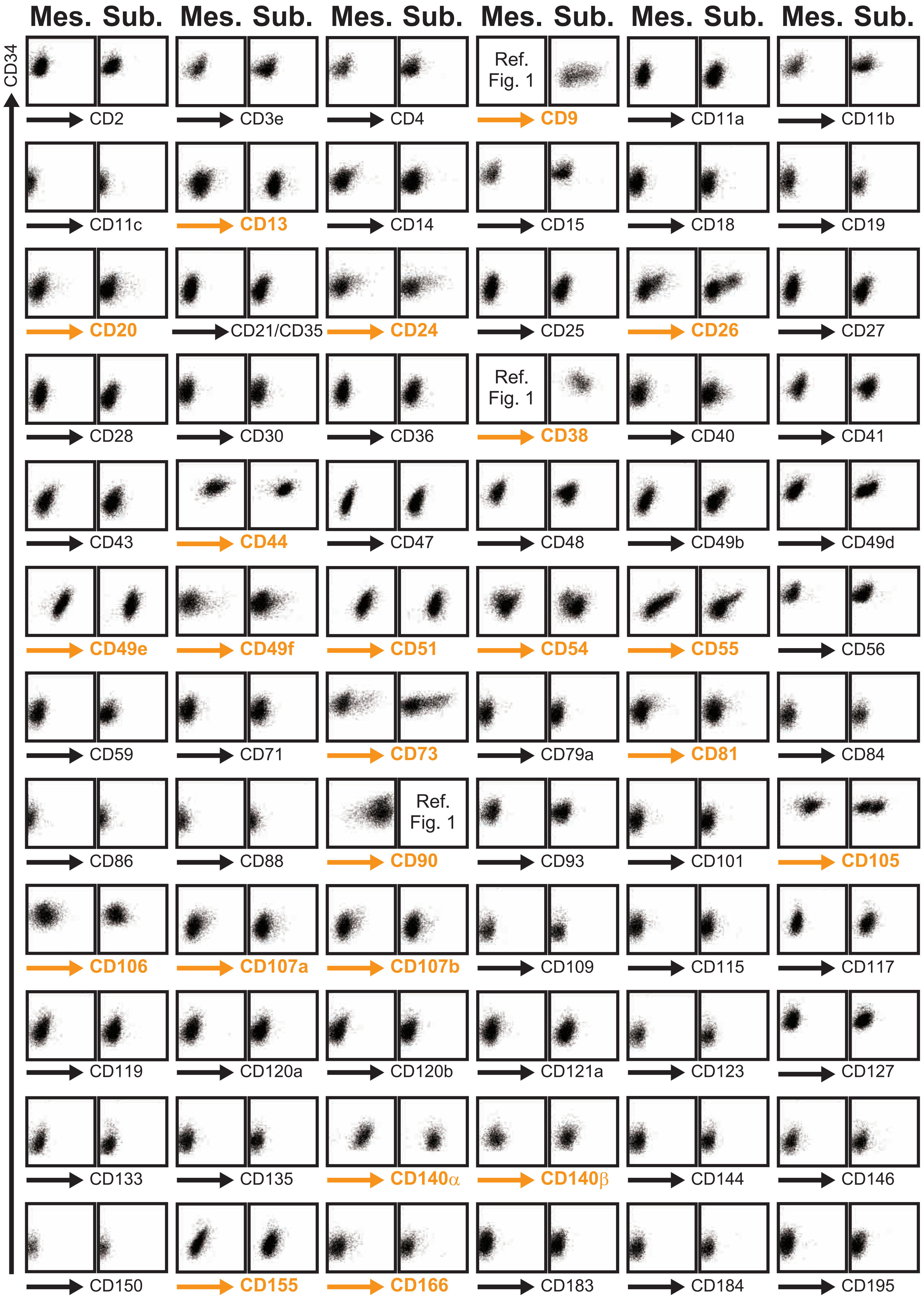
Mes. Sub. Mes. Sub. Mes. Sub. Mes. Sub. Mes. Sub. Mes. Sub.
CD34
CD2 CD3e CD4 CD9 CD11a CD11b
Ref. Fig. 1
CD11c CD13 CD14 CD15 CD18 CD19
CD20 CD21/CD35 CD24 CD25 CD26 CD27
CD28 CD30 CD36 CD38 CD40 CD41
CD43 CD44 CD47 CD48 CD49b CD49d
CD49e CD49f CD51 CD54 CD55 CD56
CD59 CD71 CD73 CD79a CD81 CD84
CD86 CD88 CD90 CD93 CD101 CD105
CD106 CD107a CD107b CD109 CD115 CD117
CD119 CD120a CD120b CD121a CD123 CD127
CD133 CD135 CD140α CD140β CD144 CD146
CD150 CD155 CD166 CD183 CD184 CD195

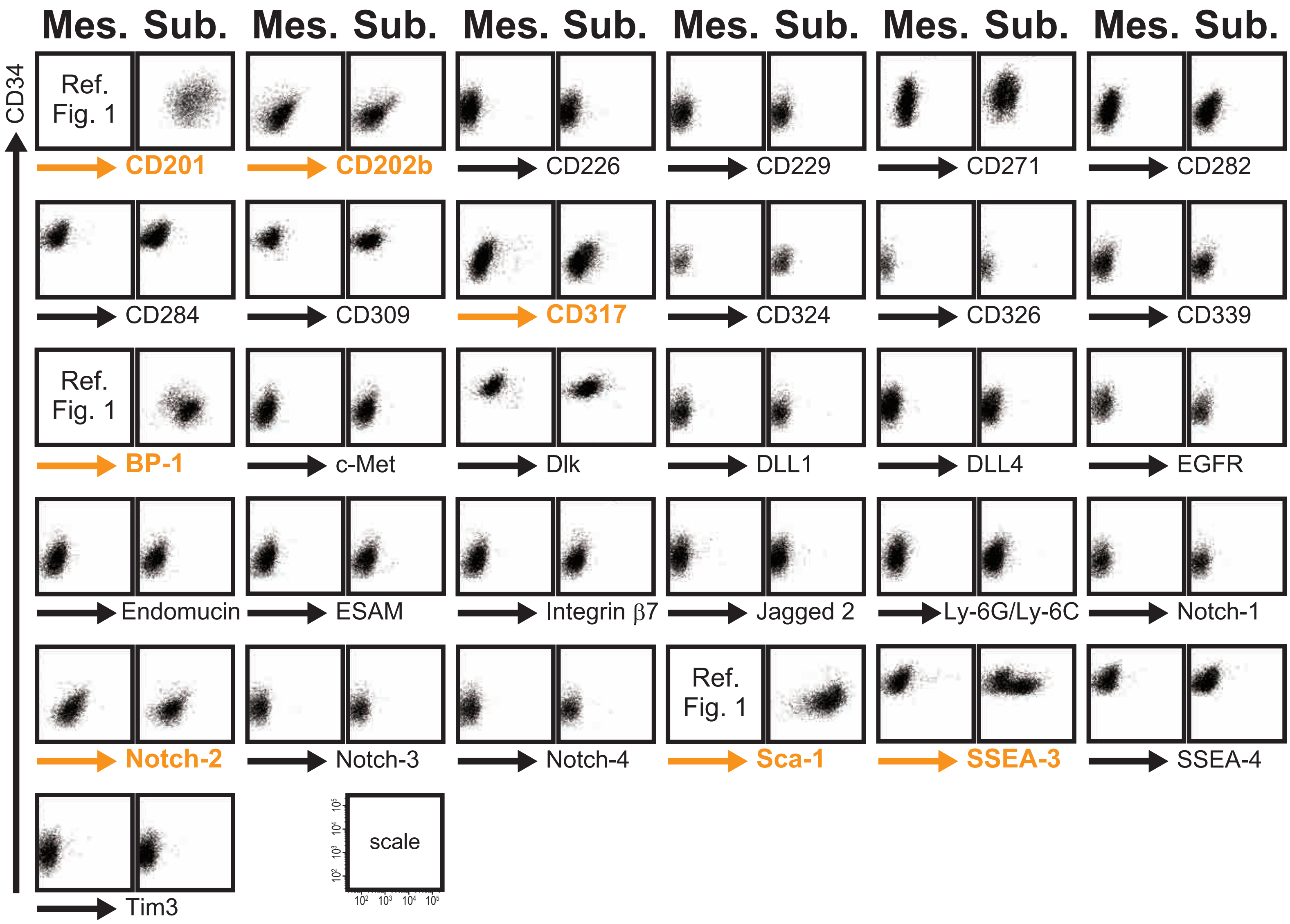

Mes. Sub. Mes. Sub. Mes. Sub. Mes. Sub. Mes. Sub. Mes. Sub.
CD34
Ref. Fig. 1
CD201
CD202b
CD226
CD229
CD271
CD282
CD284
CD309
CD317
CD324
CD326
CD339
Ref. Fig. 1
BP-1
c-Met
Dlk
DLL1
DLL4
EGFR
Endomucin
ESAM
Integrin β7
Jagged 2
Ly-6G/Ly-6C
Notch-1
Notch-2
Notch-3
Notch-4
Ref. Fig. 1
Sca-1
SSEA-3
SSEA-4
Tim3
scale

Supplementary Figure 1 (1-1 and 1-2)

The expression profile of all the surface antigens listed in Table 1 in SVF cells of mesenteric and subcutaneous WATs

The fraction of Lin^{-} $CD29^{+}$ $CD34^{+}$ cells is shown in the dot plot pictures. The gating hierarchy of Lin^{-} $CD29^{+}$ $CD34^{+}$ cells is shown in Fig. 1. The surface antigens expressed in mesenteric or subcutaneous WAT are shown in orange. The other surface antigens showed similar dot plot patterns to those of isotype controls (Supplemental Dataset S1).

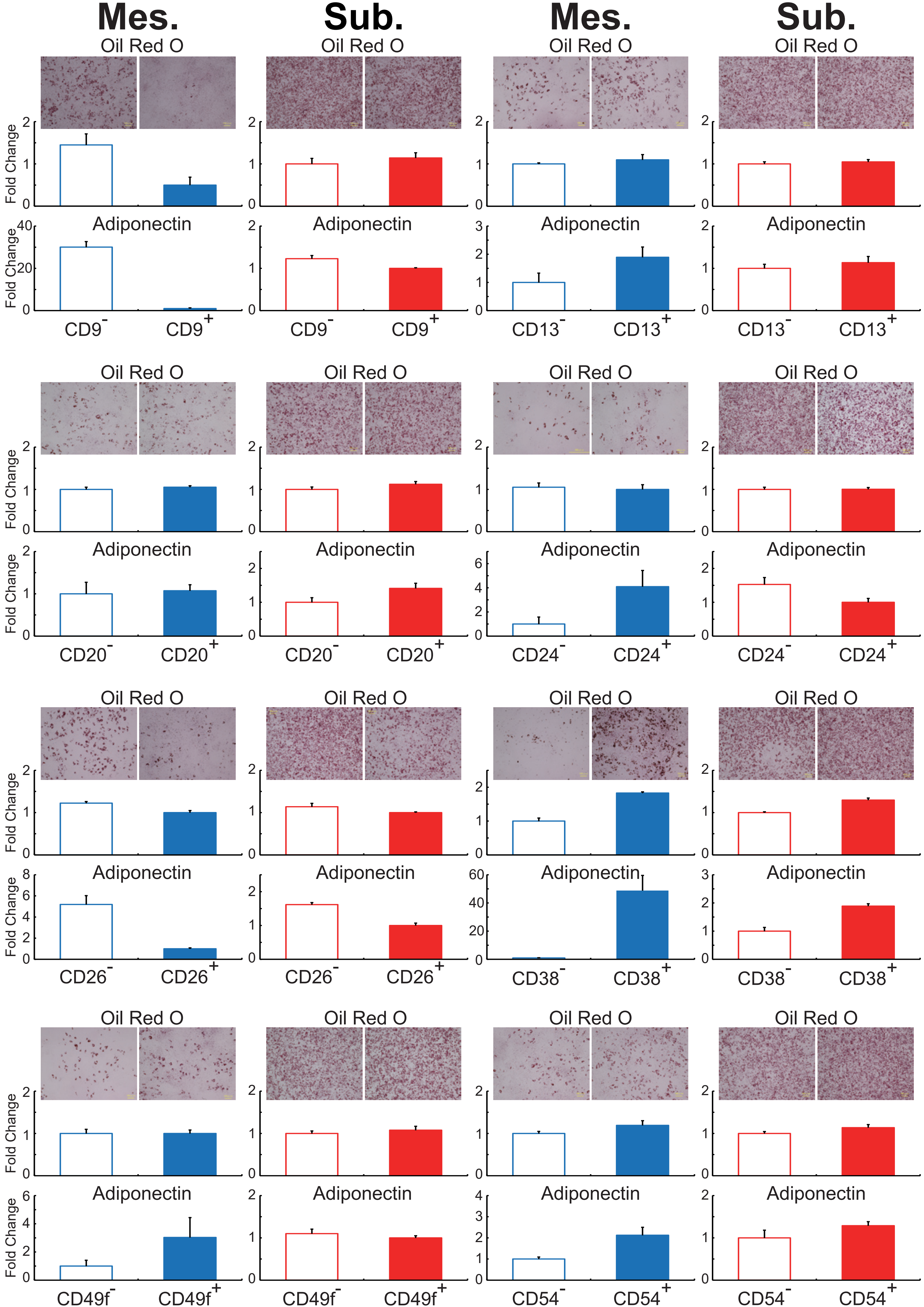

Mes.
Sub.
Mes.
Sub.
Oil Red O
Adiponectin
Fold Change
CD9-
CD9+
CD13-
CD13+
CD20-
CD20+
CD24-
CD24+
CD26-
CD26+
CD38-
CD38+
CD49f-
CD49f+
CD54-
CD54+

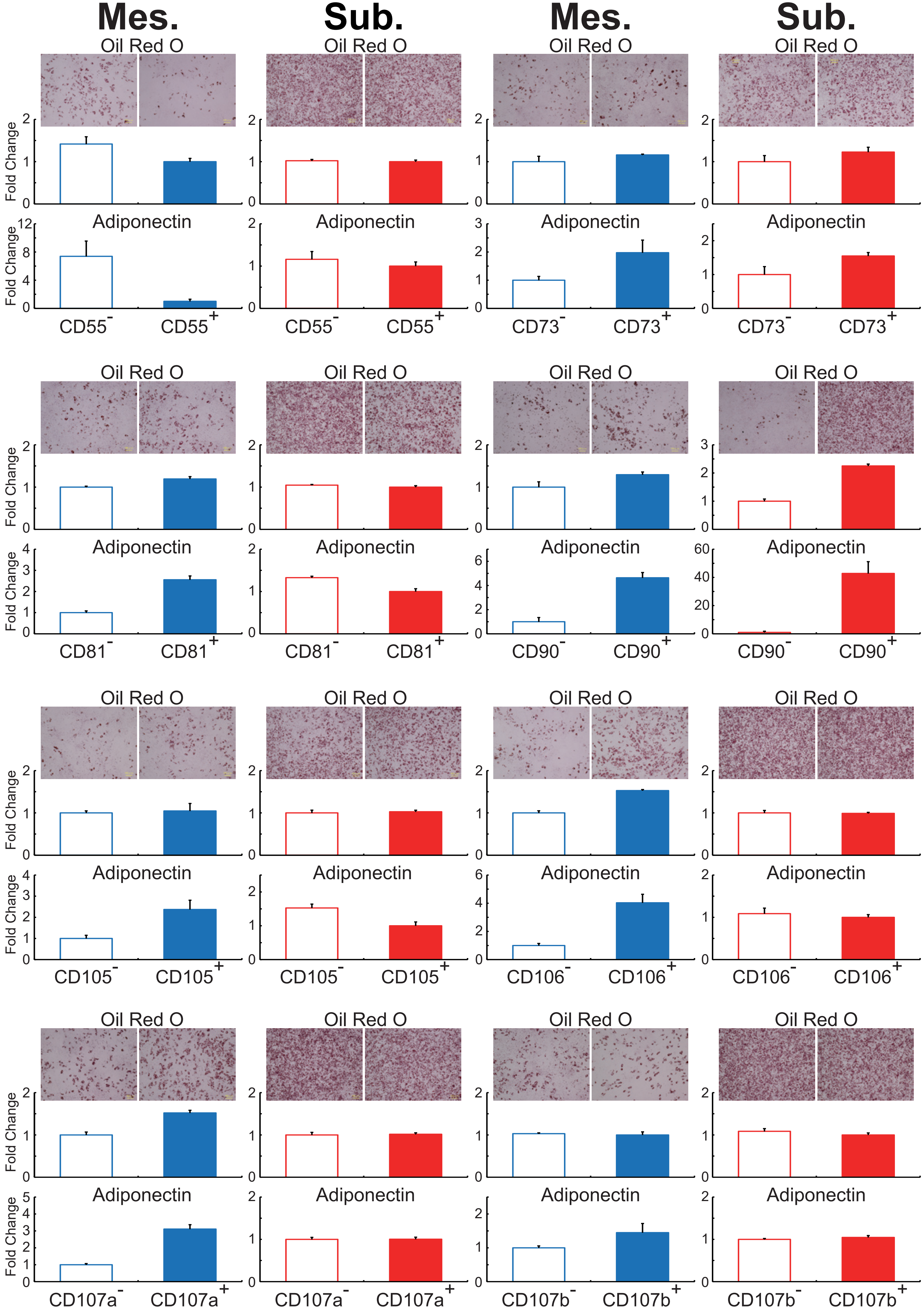
Mes.
Sub.
Mes.
Sub.
Oil Red O
Fold Change
Adiponectin
CD55-
CD55+
CD73-
CD73+
CD81-
CD81+
CD90-
CD90+
CD105-
CD105+
CD106-
CD106+
CD107a-
CD107a+
CD107b-
CD107b+

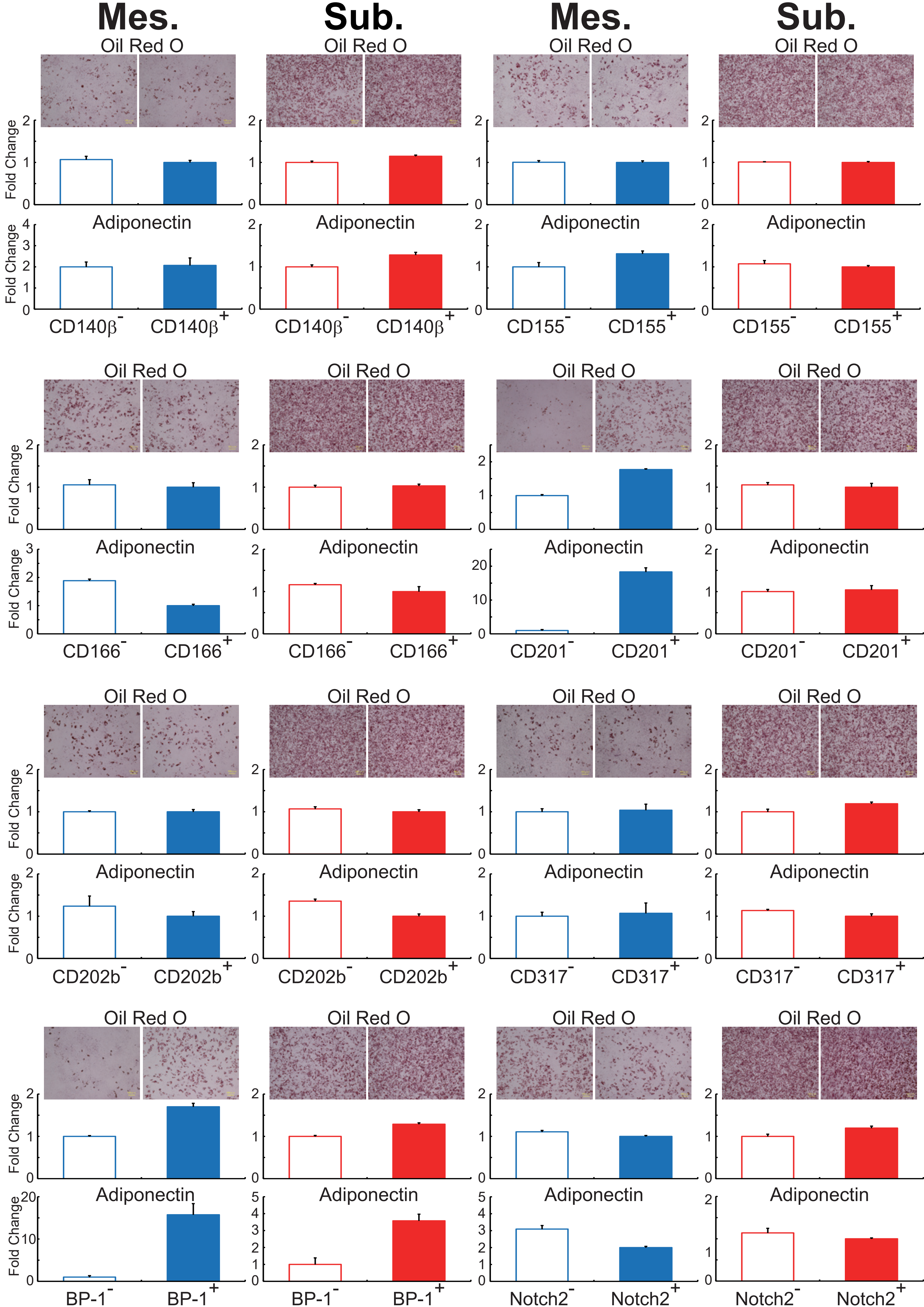

Mes.
Sub.
Mes.
Sub.
Oil Red O
Adiponectin
Fold Change
CD140β-
CD140β+
CD155-
CD155+
CD166-
CD166+
CD201-
CD201+
CD202b-
CD202b+
CD317-
CD317+
BP-1-
BP-1+
Notch2-
Notch2+

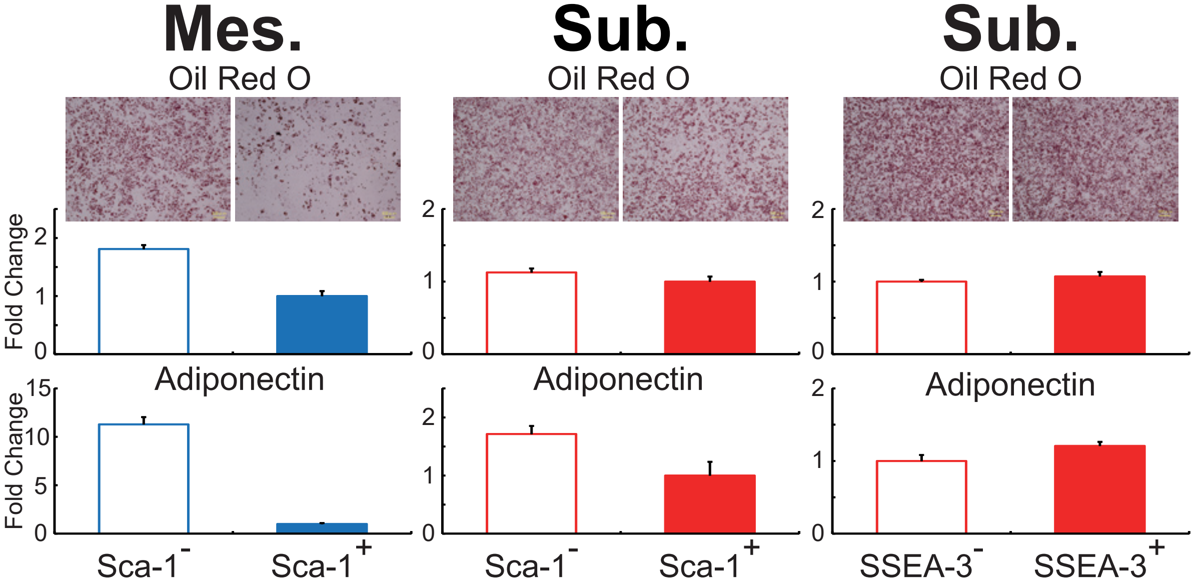

Mes.
Sub.
Sub.
Oil Red O
Oil Red O
Oil Red O
Fold Change
Adiponectin
Adiponectin
Adiponectin
Fold Change
Sca-1-
Sca-1+
Sca-1-
Sca-1+
SSEA-3-
SSEA-3+

Supplementary Figure 2 (2-1, 2-2, 2-3, and 2-4)

In vitro screening for candidate surface markers of mesenteric and subcutaneous *in vitro* adipogenic cells

SVF cells from mesenteric and subcutaneous WATs were sorted, cultured and after reaching 100% confluency, treated with adipogenic differentiation medium. The medium was replaced with DMEM containing 10% FBS 2 days after commencement of differentiation; after 48 h, the medium was collected and Oil Red O staining was performed. The adiponectin concentrations in the medium were assessed. Scale bars, 100 µm. The values are expressed as the means ± SD. n = 3-5 in each group.

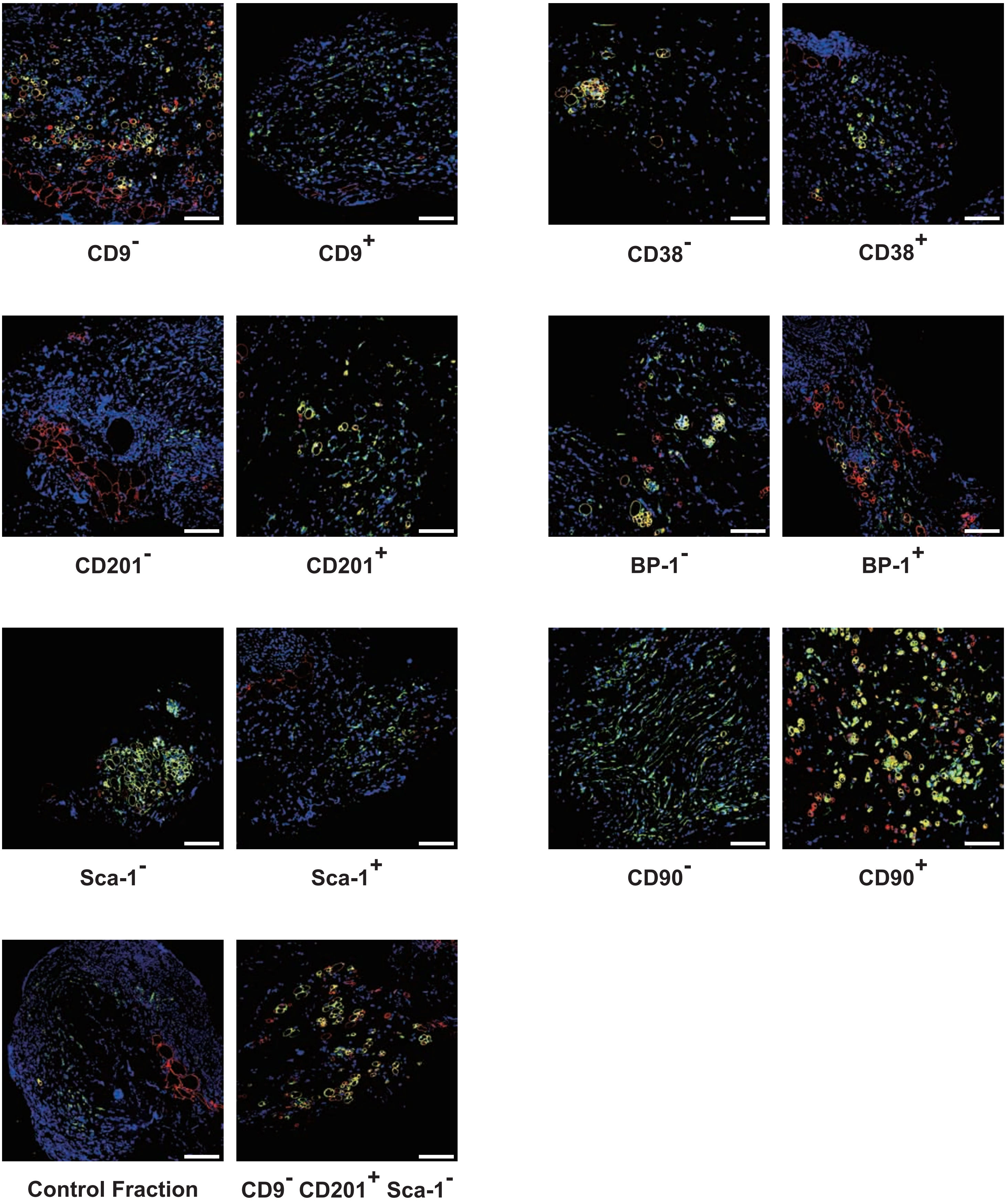
CD9^{-}
CD9^{+}
CD38^{-}
CD38^{+}
CD201^{-}
CD201^{+}
BP-1^{-}
BP-1^{+}
Sca-1^{-}
Sca-1^{+}
CD90^{-}
CD90^{+}
Control Fraction
CD9^{-} CD201^{+} Sca-1^{-}

Supplementary Figure 3

Merged images related to Fig.4 and Fig. 5d with DAPI staining

GFP, perilipin, and nuclei are shown in green, red, and blue, respectively. Scale bars, 100 µm.

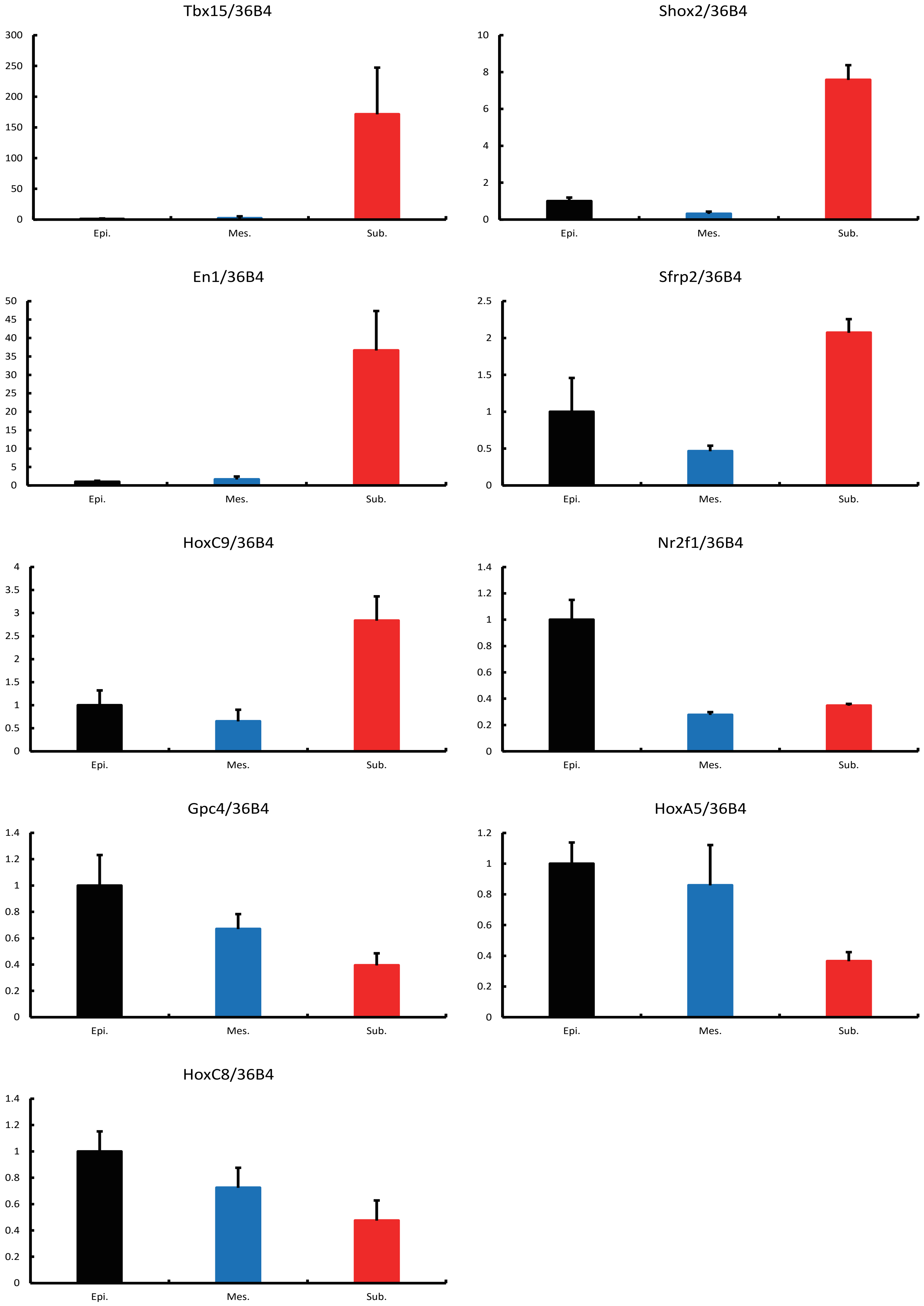
Tbx15/36B4
300
250
200
150
100
50
0
Epi.
Mes.
Sub.
Shox2/36B4
10
8
6
4
2
0
Epi.
Mes.
Sub.
En1/36B4
50
45
40
35
30
25
20
15
10
5
0
Epi.
Mes.
Sub.
Sfrp2/36B4
2.5
2
1.5
1
0.5
0
Epi.
Mes.
Sub.
HoxC9/36B4
4
3.5
3
2.5
2
1.5
1
0.5
0
Epi.
Mes.
Sub.
Nr2f1/36B4
1.4
1.2
1
0.8
0.6
0.4
0.2
0
Epi.
Mes.
Sub.
Gpc4/36B4
1.4
1.2
1
0.8
0.6
0.4
0.2
0
Epi.
Mes.
Sub.
HoxA5/36B4
1.2
1
0.8
0.6
0.4
0.2
0
Epi.
Mes.
Sub.
HoxC8/36B4
1.4
1.2
1
0.8
0.6
0.4
0.2
0
Epi.
Mes.
Sub.

Supplementary Figure 4

mRNA expression analysis of the genes that were expressed predominantly in epididymal or subcutaneous WAT (Ref. 32)

Comparison of expression patterns of the epididymal or subcutaneous WAT-specific genes were performed among epididymal, mesenteric, and subcutaneous WATs.
